# DNA methylation profiles reveals STAB1‐mediated endothelial cell and immune cell interactions in Moyamoya disease

**DOI:** 10.1002/ctm2.70367

**Published:** 2025-06-03

**Authors:** Shihao He, Zhenyu Zhou, Rui Liang, Chengxu Lei, Yutong Liu, Jialong Yuan, Youjia Tang, Yuanli Zhao

**Affiliations:** ^1^ Department of Neurosurgery Peking Union Medical College Hospital Peking Union Medical College and Chinese Academy of Medical Sciences Beijing China; ^2^ Department of Neurosurgery Beijing Tiantan Hospital Capital Medical University Beijing China; ^3^ Jiujiang City Key Laboratory of Cell Therapy The First Hospital of Jiujiang City Jiujiang China; ^4^ Department of Neurosurgery The First Hospital of Jiujiang City Jiujiang China

1

Dear editor

This investigation provides a detailed characterisation of the multifaceted pathological processes driven by DNA methylation in Moyamoya disease (MMD). The Stabilin‐1 (STAB1)‐mediated interaction between endothelial and immune cells modulates vascular homeostasis, potentially contributing to MMD pathogenesis.

MMD is a chronic cerebrovascular disease which is characterised with distal stenosis of internal carotid artery.^1^ Yet the marked clinical heterogeneity of Moyamoya disease suggests that genetic variants alone are insufficient to explain its pathogenesis, underscoring the need to interrogate complementary epigenetic mechanisms such as DNA methylation. Sung et al. employed the Illumina 450K array on a small cohort of endothelial cells and validated their findings in a HUVEC tube formation assay, revealing overexpressed Sort1's role in MMD angiogenesis.^2^ However, their limited sample size and exclusive focus on endothelial cells constrain the generalisability of these results, underscoring the need for broader investigation. Tokairin et al. used the Illumina 850 K to conduct genome‐wide DNA methylation in female cohorts. In MMD, hypomethylation was observed in genes regulating natural killer cell signalling, cell growth and migration, DNA methylation and so on.^3^ The study did not stratify by clinical subtype, lacked male participants, and provided no functional validation. In contrast, the current work employs a balanced, subtype‐defined discovery set, couples methylome data with intracranial RNA‐seq, and experimentally demonstrates that STAB1 hypomethylation enhances endothelial ECM production and tube formation. These additions not only corroborate Tokairin's immune‐inflammatory theme but also position STAB1 as a mechanistic link between aberrant immunity and intimal thickening in Moyamoya disease.

In the discovery cohort, we enrolled 30 participants, comprising 10 with haemorrhagic (HEM) and 10 with ischemic (IS) Moyamoya disease, alongside 10 healthy controls (HC). We mapped the DNA methylation profiles of ischemic and haemorrhagic Moyamoya disease patients via the Illumina 850K chip (Figure S1). The middle cerebral artery (MCA) sequencing data of 11 MMD patients and 9 control patients from GSE157628 were used as the validation cohort. Through weighted gene co‐expression network analysis (WGCNA) and differential analysis of the discovery cohort and the validation cohort, the most potential biomarker, STAB1, was obtained (Figures S1, S2 and 1A).

**FIGURE 1:**
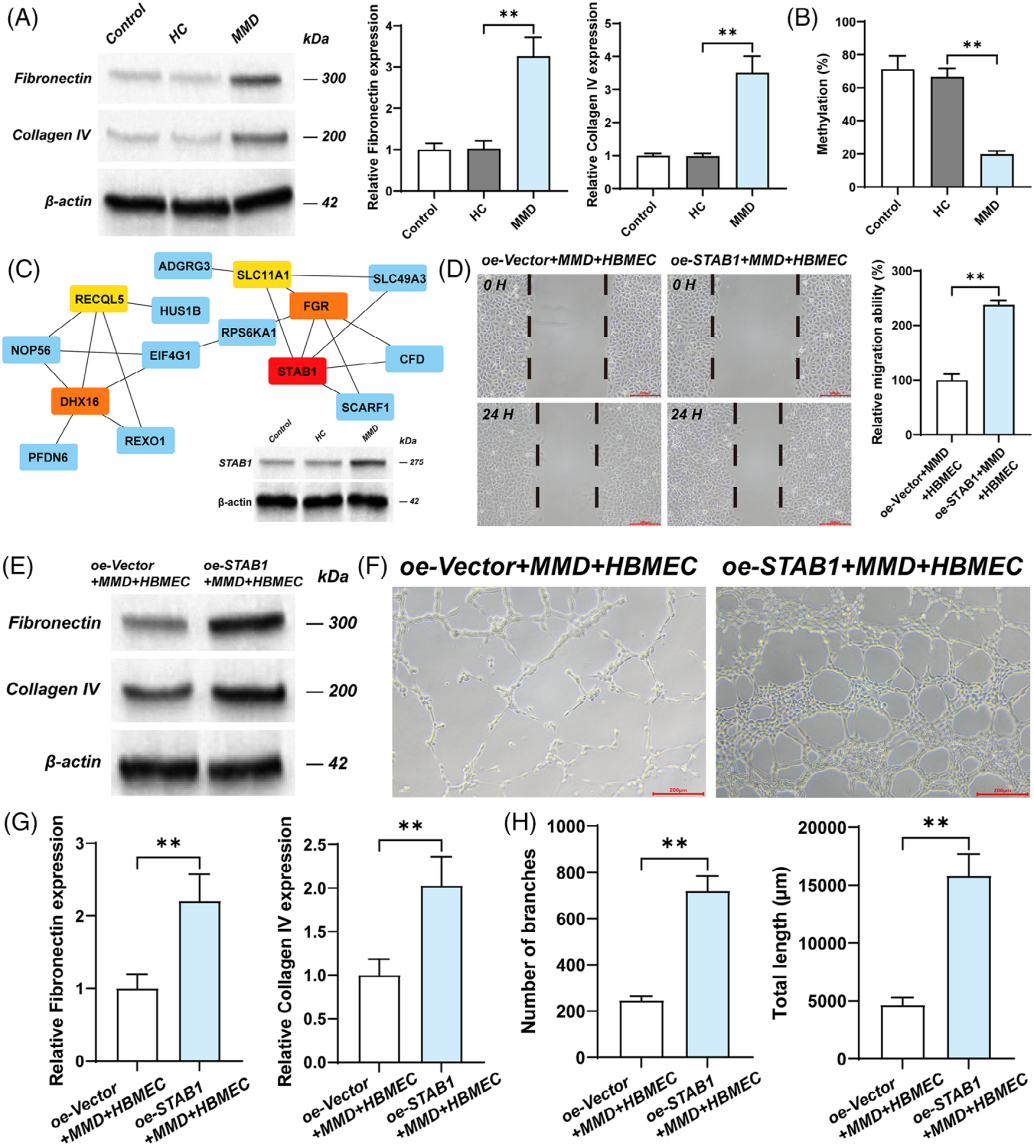
The effect of overexpression of STBA1 on endothelial cells. (A) The PPI network presents the connections among methylation‐related differential genes. Blue nodes signify regular nodes, while non‐blue nodes indicate hub genes. The closer the colour is too red, the higher the MCC score. (B) The Western Blot results indicate the expression level of STAB1 in HBMECs stimulated by serum. (C) The figures and bar charts demonstrate the effect of overexpression of STAB1 on the tube‐forming ability of HBEMCs. Compared to the oe‐Vector + MMD + HBMEC group, the number and length of branches in the oe‐STAB1 + MMD + HBMEC group are significantly increased (*p < .05; **p < .01); MMD+ HBMEC: HBMECs cultured with the serum of MMD patients). (D) The figures and bar charts illustrate the effect of overexpression of STAB1 on the migratory ability of HBEMCs. Compared to the oe‐Vector + MMD + HBMEC group, the migratory ability of the oe‐STAB1 + MMD + HBMEC group is significantly enhanced (*p < .05; **p < .01); MMD+ HBMEC: HBMECs cultured with the serum of MMD patients.

The extracellular matrix (ECM) is a macromolecular reticular structure synthesised and secreted by cells to the extracellular space. Matsuo et al. conducted immunohistochemical analysis on the specimens of the middle cerebral arteries (MCA) from autopsies of MMD patients and found that ECM was significantly accumulated in the thickened intima.^4^ The ECM components (fibronectin, collagen IV) were also found to be upregulated in ECs cultured with MMD serum and overexpressing STAB1 EC (Figure 1A, E, and G). This suggests that STAB1 may promote the thickening of the vascular intima by enhancing the synthesis of ECM by ECs. Ye et al. explored the effect of RNF213 on human brain microvascular endothelial cells (HBMEC) in Moyamoya disease.^5^ They found that RNF213 knockdown promoted the proliferation, migration and tube formation of HBMEC. This suggests that there is a complex relationship between endothelial cell dysfunction and pathological angiogenesis in Moyamoya disease. In this study, to demonstrate the genetic and functional characteristics of EC in MMD patients, human brain microvascular endothelial cells were cultured with the serum of patients with Moyamoya disease as the cell model for in vitro experiments. Adachi et al. reported the influence of STAB1 on the tube‐forming ability of endothelial cell (EC) through a tube formation assay.^6^ We found that overexpressed STAB1 promoted the tube formation and migration capabilities of EC (Figures S2 and 1D, F, and H). Therefore, in the pathogenesis of MMD, overexpressed STAB1 may disrupt vascular homeostasis by acting on EC. STAB1 mediates immune cell adhesion to the vascular wall and directed chemotaxis.^7^ Furthermore, the GSEA results suggest that among the pathways enriched by STAB1, there are several related to immunity, such as Antigen processing and presentation, Intestinal immune network for IgA production (Figure S4). The result of immune infiltration analysis suggests that STAB1 is correlated with various immune cells, of which Treg and CD56^bright^NK cells are more significant than other (Figure 3A and B).

Regulation T cell (Treg cell) are a unique cell lineage within the adaptive immune microenvironment that has been reported to impact on angiogenesis.^8^ Weng et al. found that the proportion of Treg cells in the peripheral blood of patients with MMD was significantly higher than the other groups.^9^ We also discovered an increased number of Treg cells in MMD, which might be associated with ischemia and hypoxia induced by vascular stenosis. In our preliminary direct‐contact co‐culture, Tregs appeared to enhance endothelial‐cell proliferation (Figure 2D). In MMD, EC may recruit Tregs due to hypoxia, which reach the hypoxic area and affect angiogenesis by regulating EC.

**FIGURE 2:**
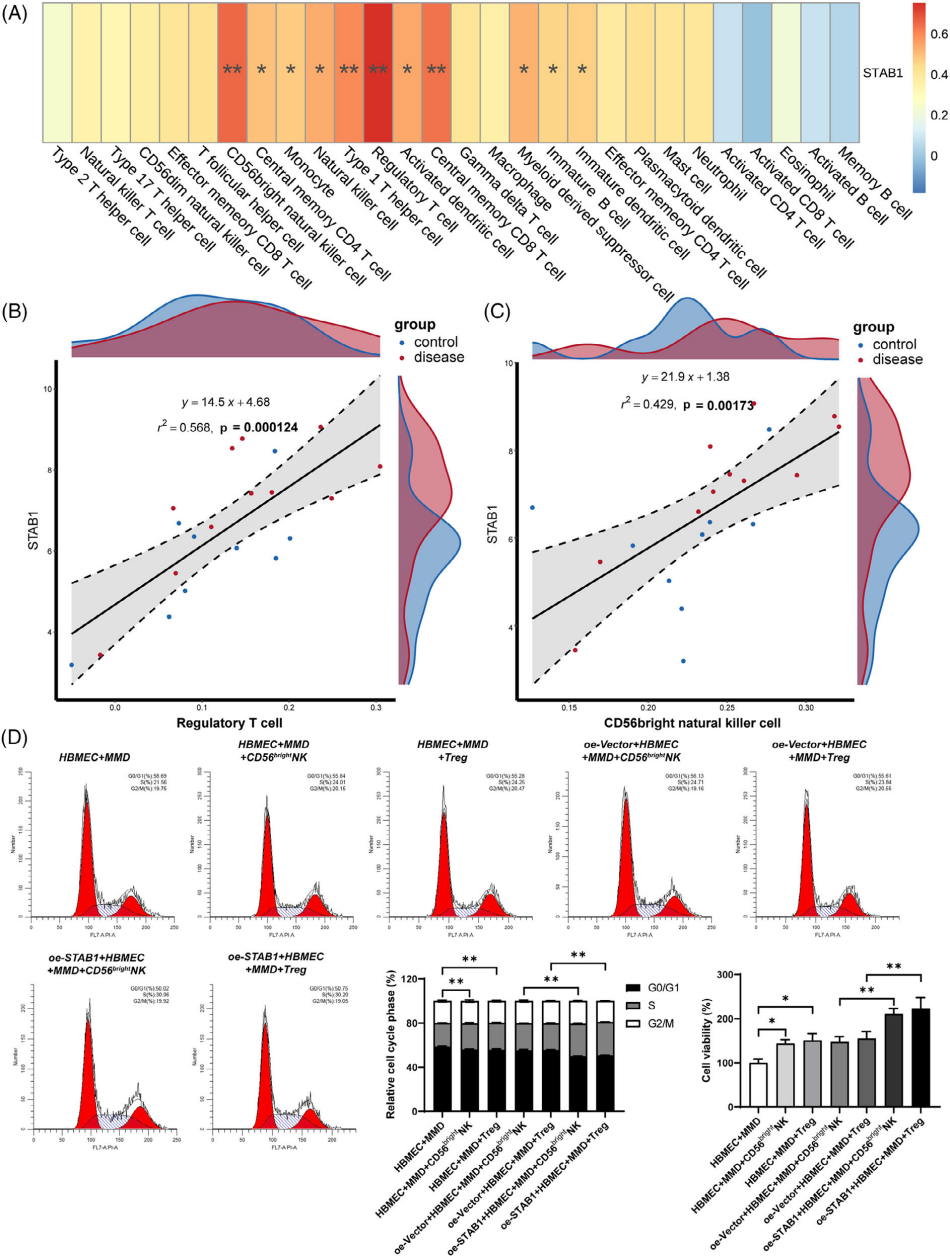
Overexpression of STAB1 augments the proliferative capacity of HBMEC in conjunction with immune cells. (A) The heatmap presents the correlation between STAB1 and immune cells. Red indicates a positive correlation, while blue indicates a negative correlation (*p < .05; **p < .01). (B) Correlation analysis between STAB1 and Treg cells. Blue represents the control group, and red represents the disease group. (C) Correlation analysis between STAB1 and CD56^bright^NK cells. Blue represents the control group, and red represents the disease group. (D) Flow cytometry was employed for the analysis of the cell cycle in each group of HBMEC. The left bar chart depicts the proportion of each group of HBMEC in the GO/G1, S, and G2/M phases of the cell cycle. The right bar chart showcases the proliferative capacity of each group of HBMEC (*p < .05; **p < .01).

CD56^bright^ nature killer cell (CD56^bright^NK cell) is a subset of nature killer cells. Li et al. reported the cytotoxic effect of NK cells around the lesion on cerebrovascular endothelial cells in intracerebral haemorrhage.^10^ In our study, an interaction between CD56 ^bright^NK cell and the abnormally enlarged cytoskeleton of ECs was likewise observed (Figures 3A and D and 4, S5, and S6). In MMD, it is possible that the enlarged cytoskeleton of endothelial cells induces the proliferative promotion effect of CD56^bright^NK cell on endothelial cells. STAB1 mediates immune cell adhesion to EC for immune response and inflammation.^7^ Overexpression of STAB1 in EC may attract CD56 ^bright^NK and Treg cells to the area for regulating angiogenesis. Interestingly, this indicates that there may be a positive interaction between EC, CD56 ^bright^NK and Treg cell. This positive interaction may accelerate and amplify the vascular stenosis and collateral proliferation, as well as the inflammation in MMD.

**FIGURE 3:**
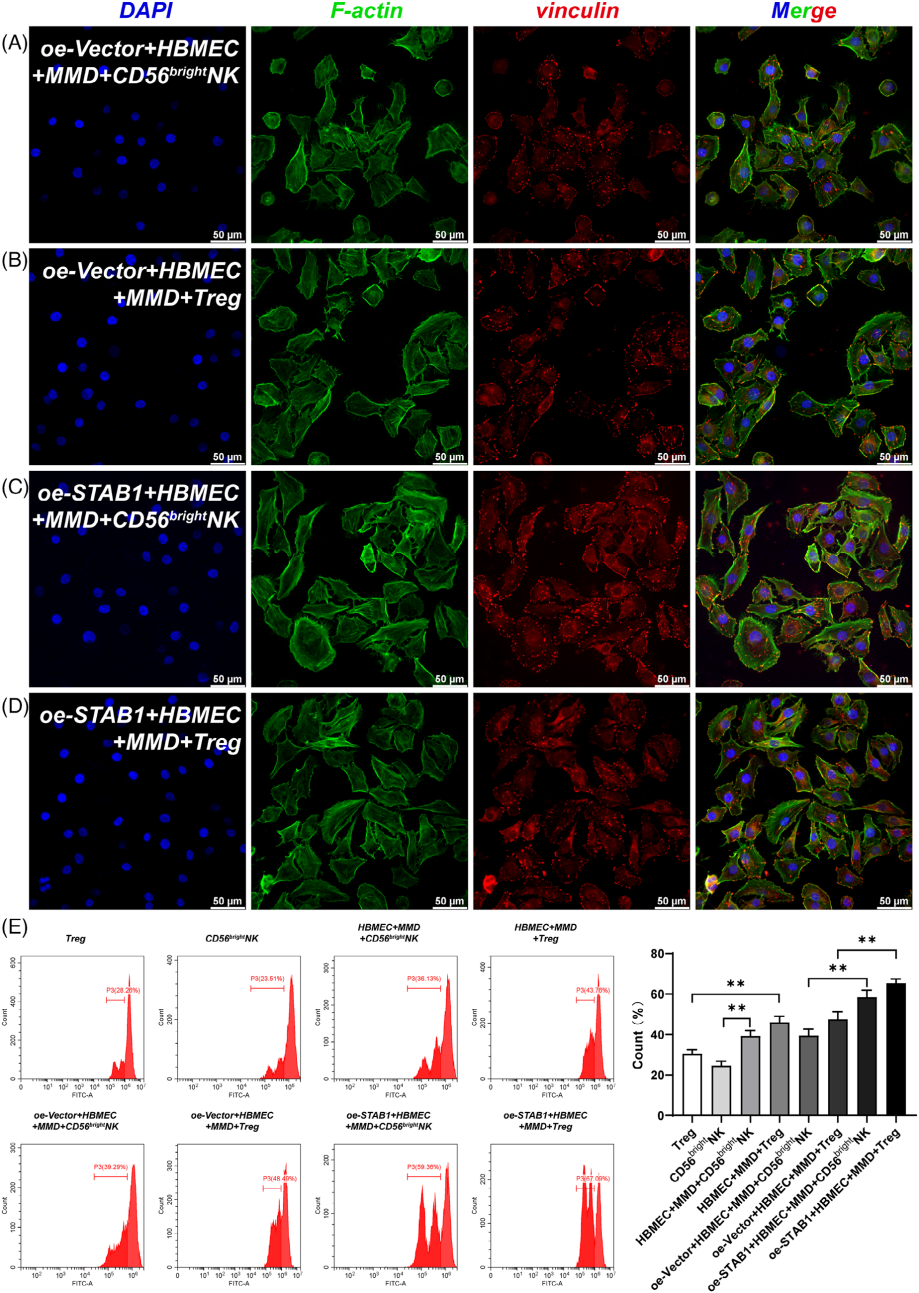
Interactions between HBMEC and immune cells mediated by STAB1 overexpression. (A–D) Immunofluorescence staining was performed on HBMEC after plasmid transfection and co‐culture. Blue (DAPI): nucleus. Green (F‐actin): cytoskeleton. Red (vinculin): adherens junction. Scale bar: 50 µm. (E) After co‐culture, the cell proliferation ability of Treg and CD56^bright^NK cells was determined. The bar chart presents the cell proliferation ability of each group (*p < .05; **p < .01).

**FIGURE 4:**
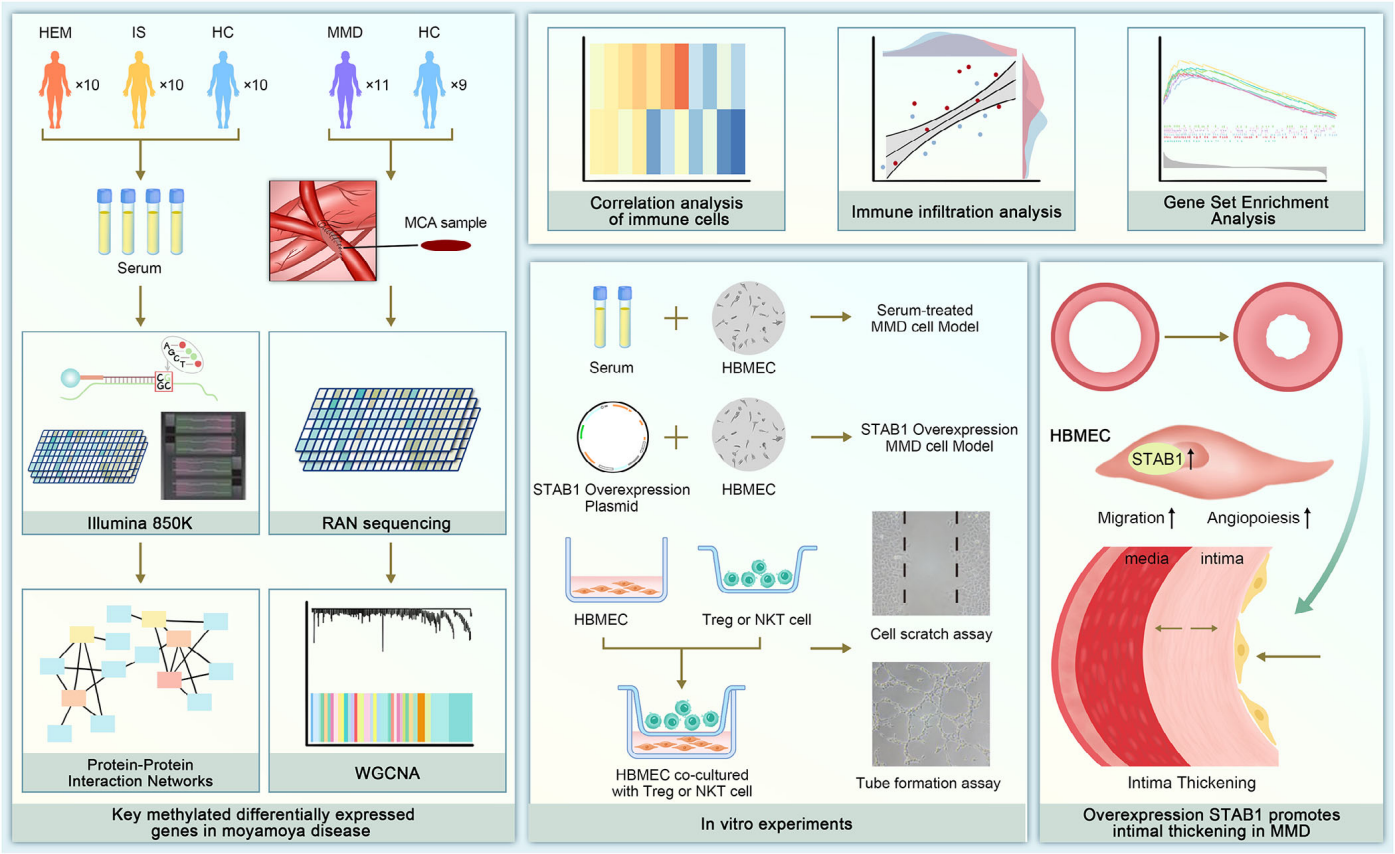
Mechanism of DNA methylation regulating STAB1 that promotes intimal thickening in Moyamoya disease. In the discovery cohort, DNA methylation analysis of the serum was conducted using the Illumina 850k chip. In the validation cohort, transcriptomic profiling of middle‐cerebral‐artery specimens (11 MMD, 9 controls) was performed with the Agilent SurePrint G3 Human GE v2 8 × 60 K one‐colour microarray (GSE157628). By integrating differential analysis, WGCNA, and PPI, STAB1 was identified as a candidate potential biomarker. Through bioinformatics analysis and in vitro experiments, it was found that overexpression of STAB1 promotes intimal thickening in MMD by regulating EC.

While we mitigated this limitation by validating differentially methylated genes in independent arterial transcriptome datasets and conducting functional analyses in endothelial cells, the lock‐specific methylation changes within the vessel wall still need to be confirmed by expanding the sample size and in vivo studies. In vitro experiments, co‐culture experiments lack transwell control and cytokine secretion profiles.

In conclusion, our study provides valuable implications for the DNA methylation in MMD, pushing the boundaries of our knowledge of the unclear pathogenesis.

## AUTHOR CONTRIBUTIONS

SH, YJ, and YL conceived and designed the experiments. SH, ZY, CX, LR, YT, and JZ performed experiments. YJ and YL contributed reagents, materials, and analytical tools. SH, ZY, YJ, and YL wrote the manuscript.

## CONFLICT OF INTEREST STATEMENT

The authors declare no competing interests.

## FUNDING INFORMATION

This study was supported by National High Level Hospital Clinical Research Funding (2023‐PUMCH‐E‐011), CAMS Innovation Fund for Medical Sciences (CIFMS) (2023‐I2M‐C&T‐B‐048). The above funds provide the testing and processing costs, data collection, analysis and interpretation of this experiment.

## ETHICS STATEMENT

This study was approved by the Institutional Ethics Committee of Peking Union Medical College Hospital, Beijing, China (I‐24PJ1573). All participants signed informed consent forms.

## Supporting information



Supporting Information

## Data Availability

Any information presented in the current study are available from the corresponding author on reasonable request.
