# Supplementary material for: DNA methylation profiles reveals STAB1‐mediated endothelial cell and immune cell interactions in Moyamoya disease
Source: Clin Transl Med. 2025 Jun 3;15(6):e70367. doi: 10.1002/ctm2.70367 (PMC12134390; doi:10.1002/ctm2.70367)
Supplement: Supplementary file 1 — Supporting Information [file CTM2-15-e70367-s001.pdf]

Supplementary materials

DNA Methylation Profiles Reveals STAB1-Mediated Endothelial cell and Immune Cell Interactions in Moyamoya Disease

Shihao He,[1,†,*] MD, PhD; Zhenyu Zhou,[4,†] MD; Rui Liang,[2, 3†] MD; Chengxu Lei,[1] MD; Yutong Liu,[1] MD; Jialong Yuan,[1] MD; Youjia Tang[*],[2, 3] MD, PhD; Yuanli Zhao[*],[1] MD, PhD;

Method

Participants

In this study, 10 patients with hemorrhagic moyamoya disease, 10 patients with ischemic moyamoya disease, and 10 healthy controls were enrolled as the discovery cohort. (all participants aged > 18 years) All participants were diagnosed according to the guidelines for MMD. All participants signed a written informed consent. Download GSE157628 from NCBI GEO (http://www.ncbi.nlm.nih.gov/geo/). Select the samples of 11 moyamoya disease patients (disease) and 9 control group samples (control) from it. Detection platform: GPL16699Agilent-039494 SurePrint G3 Human GE v2 8x60K Microarray 039381 (Feature Number version). Use limma for correction (version 3.5.8.1, https://bioconductor.org/packages/release/bioc/html/limma.html).
For different probe mapping to the same gene, the average value is taken as the expression value of the gene.

DNA extraction from experimental specimens

The DNA samples were prepared for amplification and array hybridization, which included DNA fragmentation and DNA precipitation. The ratio of the absorbances at 260 nm and 280 nm of all samples ranged from 1.7 to 2.0, the concentration was ≥ 50 ng/μL, and the total amount was ≥ 2 μg. All samples were dissolved in Tris-EDTA before preparation and were stored in a □80 ° C Forma 700 ultra-low temperature refrigerator . Use the EZ DNA Methylation-GoldTM Kit (D500550 rxns) to treat the DNA sample with bisulfite modification.

Illumina 850K chip hybridization and data quality control

The Illumina Infinium MethylationEpic BeadChip was used for hybridization with the extracted DNA fragments with INFINIUM Methylation EPIC (32 Sample) （WG-317-

1002）. After chip cleaning, single base extension, and staining, the Illumina ISCAN system was used for chip scanning. During data scanning and input, the 850K chip performs the necessary quality control for staining and extension for each sample to ensure that the experimental procedures and the chip are used correctly.

The Illumina 850K chip encompasses two types of probes: Infinium I and Infinium II. In Infinium I, each methylation site is devised with two probes: M-type magnetic beads for the detection of methylated sites (C), and U-type magnetic beads for the detection of unmethylated sites (T). In accordance with the principle of single-base extension, only when the last base of the probe matches the template can the fluorescently labeled ddNTP be incorporated and the fluorescence signal be detected. The methylation value is computed based on the outcomes of the M and U fluorescence signals. Infinium II employs only one type of magnetic bead, and the degree of methylation at the target site is calculated based on the signal value of the extended A or G base (which respectively correspond to unmethylated and methylated sites).

For each sample and each CpG site, there is a corresponding detection P value. The smaller the P value, the more reliable the information at the site. If more than 10% of the CpG sites in a sample have detection P values greater than 0.01, the sample is considered to be filtered out; If a sample is filtered out, and a CpG site still has a detection P value greater than 0.01, the site is also filtered out; In this study, no samples were filtered out, and 6221 CpG sites were filtered out. In the Illumina 850k chip, each CpG site is represented by a probe that is distributed across multiple magnetic beads (Beads). For each sample and CpG site, there is an NBeads value, which represents the number of beads that produce a fluorescence signal. The larger this value, the more reliable the probe signal. If the NBeads value for a CpG site in more than 5% of the samples is less than 3, the site is excluded; This batch of data excluded 9325 sites. In the 850k chip, there are a large number of control probes for non-CpG detection, such as 59 SNP sites, 635 various control probes, etc., which should be excluded in EWAS analysis. This batch of data has 2,878 excluded SNP sites. Because methylation chip is essentially an SNP chip, SNP polymorphisms can affect DNA methylation detection. Therefore, this analysis filters out CpG sites with MAF>0.05 in the EAS. 10560 sites were filtered out in this batch of data. According to research, certain CpG sites corresponding to probes can blast to multiple different chromosomal regions. These probes will not accurately determine the methylation level at the designed site. Therefore, these CpG sites should be filtered out. This batch of data has 8,468 filtered sites. When conducting an EWAS analysis, if the trait of interest is unrelated to sex, CpG sites on the sex chromosomes need to be filtered out. Because the methylation distribution at these sites is clearly associated with sex and can affect subsequent EWAS analysis, 186,440 sites were filtered out in this batch of data.

After filtering, the final data used for analysis consisted of 30 samples with 8,120,281 CpG sites.

Detection of differentially methylated regions

For the DMP (Differentially Methylated Positions) analysis of this project, the Benjamini & Hochberg method was used to correct for multiple testing p-values, and the final threshold of $P<0.05$ was used as the cutoff for significant differential methylation sites. Differentially methylated sites often cluster together in the genome, forming a differentially methylated region (DMR). For the DMR analysis in this project, the Benjamini & Hochberg method was used to correct for multiple testing P-values, and the final threshold of FDR-adjusted $p < 0.05$ was used as the cutoff for significant differentially methylated sites. A total of 1,304 significant differentially methylated regions were identified. Use ggplot2 (3.5.0, https://cran.r-project.org/web/packages/ggplot2/index.html) to create a volcano plot to show the differences in methylation regions. Map each DMR-corresponding DMP to the Body, TSS1500, and TSS200 regions. Genes mapped to the Body region are annotated to genebody. Genes mapped to the TSS1500 and TSS200 regions are annotated to promoter. Genes annotated as genebody and promoter are considered differentially methylated genes (DMG).

GO and KEGG enrichment analysis

R language's clusterProfiler package (4.10.0, https://bioconductor.org/packages/release/bioc/html/clusterProfiler.html) was ued to perform GO and KEGG functional analysis. Benjamini & Hochberg method was used for multiple testing correction and obtained the adjusted p-value. We screened the significant enrichment results by setting the threshold of adjust.pvalue < 0.05 and count >= 2.

GEO data differential analysis

The analysis set samples were divided into two groups, disease (experimental group) and control (control group), and differential expression analysis was performed using the limma package in R language. Genes with a p-value < 0.05 and $|log2FC| > 0.263$ were selected as differential genes between moyamoya disease and control. The differentially methylated genes (DMGs) were intersected with the up-regulated differential genes of moyamoya disease and the down-regulated differential genes of moyamoya disease to obtain differentially methylated genes related to moyamoya

disease.

Protein-Protein Interaction Network

Search for the interactions between the protein products of differentially methylated gene products related to STRING (https://cn.string-db.org/) and construct a PPI network. Visualize the network using Cytoscape (3.9.0) (http://www.cytoscape.org/) and calculate the key performance indicators for the sub-modules of genes: MCC. Select the top five genes with the highest scores as hub genes.

Weighted correlation network analysis

The R package WGCNA (1.61, https://cran.r-project.org/web/packages/WGCNA/) was used to perform a gene co-expression network analysis (WGCNA) on the differential gene expression matrix of moyamoya disease. By calculating the correlation between modules and phenotypes, we selected the module with the highest correlation with the disease and a p-value less than 0.05 as the disease-related module of moyamoya disease. We then obtained the differential disease-related hub genes by intersecting the disease-related module genes with the methylation-related differential genes.

Immune cell infiltration analysis

Using the ssGSEA (1.5.0.1, https://www.bioconductor.org/packages/devel/bioc/vignettes/GSVA/inst/doc/GSVA.html) method, the expression levels of the 28 immune cell infiltration scores were calculated based on the expression data of the analysis set samples. The infiltration difference between the disease and control groups was tested by t-test. Then, the correlation between the methylation-related differential disease genes and the immune cells was calculated using Pearson correlation.

Gene set enrichment analysis

Use the R package clusterProfiler to conduct a GSEA analysis on KEGG for identifying signaling pathways that are activated or inhibited in cerebral vasospasm. Filter the results with $p.adjust < 0.05$ and $|NES| > 1$ as significant results.

Cell culture and treatment

Human brain microvascular endothelial cells (HBMEC) were purchased from the Shanghai Zhong Qiao Xin Zhou Biotechnology (Shanghai, China). HBMEC are

passaged and cultivated in a tissue culture dish under the following circumstances: The ECM (1001, Shanghai Zhong Qiao Xin Zhou Biotechnology, Shanghai, China) culture medium contains 100 U/mL penicillin, 100 μg/mL streptomycin, 5% FBS (SH30070.03, Hyclone, Logan, UT, USA), and 1% ECGS (1052, Shanghai Zhong Qiao Xin Zhou Biotechnology, Shanghai, China). When the cells reach a 90% confluence, the old culture medium is discarded. The cells are washed twice with 2 mL PBS. After discarding the PBS, 2 mL of 0.25% trypsin-0.02% EDTA digestive solution is added. The cells are observed under a microscope for approximately 30 seconds. When the cells become round, 2 mL of complete culture medium is promptly added to stop the digestion. The cells are gently aspirated and collected. The cells are centrifuged at 800 rpm for 5 minutes at 4°C, and the supernatant is discarded. The cells are resuspended in complete culture medium and subcultured in flasks. The culture medium is changed every other day.

PBMC Cell sorting

Take fresh whole blood and add it to 1× dilution wash buffer in a 1:1 ratio. Dilute the blood, gently mix it, and set aside for use. Add an appropriate amount of single nucleus isolation solution to sterile centrifuge tubes. Spread the diluted blood sample evenly over the surface above the isolation solution (isolation solution: diluted whole blood = 1:2). Keep the interface between the two liquid surfaces clear. 800 g, room temperature, centrifuge for 30 minutes. After centrifugation, aspirate the plasma layer. Pipette the PBMC layer (i.e. the white membrane layer) and transfer it to a 15 mL centrifuge tube. Add 10 mL of 1L diluted washing solution to the centrifuge tube to suspend the cells. Centrifuge at room temperature for 10 minutes at 250 g, discard the supernatant.

Sorting Treg cells

Use the CD4+CD25+Treg Cell Immunomagnetic Bead Separation Kit for the sorting of Treg cells (130-091-301, Miltenyi Biotec, Bergisch Gladbach, Germany). The obtained PBMC cell suspension was counted and sorted with 107 cells as the unit dosage. CD4+T cells were obtained by negative sorting: PBMC cell suspension was centrifuged at low temperature and high speed for 300 g×10 min at 4°C, and the supernatant was discarded. Add 40 μL buffer (WB2001, NCM Biotech, SuZhou, China) for re-suspension, add 10 μL Biotin-antibody Cocktail, thoroughly mix, and incubate at 4°C in the refrigerator for 10 min away from light. Take out the cell suspension and add 30 μL buffer, 10 μLCD25-PE antibody and 20 μL anti-biotin Micro Beads, gently mix, and incubate at 4°C for 15 min away from light. Place LD sorting column in MACS separator in advance, wash the sorting column with buffer, 2 mL each time, repeat twice

(be careful not to let the buffer flow dry); The single-cell suspension was put into the sorting column, and 1 mL buffer was added when the suspension in the sorting tube quickly drained out, repeated twice, and all the outgoing suspension was collected, that is, CD4+T cells.

CD4+CD25-T cells obtained by negative sorting and CD4+CD25+T cells obtained by positive sorting: The cell suspension was centrifuged for 300 g×10 min, and the supernatant was discarded to obtain cell precipitation, then 90 μL buffer was added for re-suspension, and 10 μL anti-PE microbeads were added, gently mixed and incubated in the refrigerator at 4°C for 15 min away from light. The MS sorting column was placed on the separator in advance, the column was moisten with 0.5mL buffer, and the single-cell suspension was put into the sorting column. When the suspension in the sorting tube drained quickly, 0.5mL buffer was added, and all the outgoing suspension was collected, that is, CD4+CD25-T cells. Remove the MS sorting column from the magnetic field environment and place it in another EP tube towards

After adding 1 mL buffer to the MS separation column, the buffer was quickly squeezed into the EP tube with a piston, and the cell suspension obtained was CD4+CD25+T cells.

Treg cell culture

Anti-human CD3 monoclonal antibody and anti-human CD28 monoclonal antibody were diluted with RPMI 1640 medium, and 1.5 mL of RPMI 1640 culture containing 4μg/mL CD3 antibody and 0.5 μg/mL CD28 antibody were added to a T25 culture vial. Fill the bottom of the bottle with liquid and place the bottle flat in a refrigerator at 4 ° C overnight.

Treg cells were added into the coated culture bottle and 10 mL containing 10% FBS, 1% penicillin-streptomycin, IL-2 (final concentration: 100 ng/mL), L-glutamine (final concentration: 2 mM), sodium pyruvate (final concentration: 1 mM), β-mercaptoethanol (final concentration: 1 mm) were added. 55 mM) RPMI 1640 culture solution. After the cells continued to be cultured in the incubator for 2 days, 50% of the volume of fresh medium (containing 100 ng/mL of IL-2) was added, and the cells were transferred to the culture bottle of T75. Rehydrate every two days and transfer the cells to an uncoated culture bottle. After 14 days of culture, Treg cells were collected.

Sorting $CD56^{bright}$NK cells

The Human CD56+CD16-NK Cell Isolation Kit is used to sort $CD56^{bright}$NK cells (130-092-661, Miltenyi Biotec, Bergisch Gladbach, Germany). The obtained PBMC cell suspension was counted and sorted with 108 cells per unit. Magnetic labeling and

depletion of non-CD56 + CD16-NK cells: PBMC cell suspension was centrifuged at low temperature and high speed at 4°C for 300 g×10 min, and the supernatant was discarded. Add 400 μL buffer for re-suspension, add 100 μL CD56+ CD16-NK Cell Biotin-Antibody Cocktail, gently mix, and incubate at 4°C for 10 min away from light. Add 5 mL buffer buffer to wash the cells, centrifuge at low temperature and high speed for 300 g×10 min, and discard the supernatant. 800μL buffer was added for re-suspension, and 200μL NK Cell MicroBead Cocktail was added, and the mixture was fully mixed and incubated in 4°C refrigerator for 15 min in the dark. The cells were washed with 10 mL buffer, centrifuged at low temperature and high speed for 300 g×10 min, and the supernatant was discarded. Add 500 μL buffer for suspension. Pre-place the LD separator in the MACS separator and wash the separator with buffer, 3 mL each time, repeat twice; The single-cell suspension was put into the sorting column, and 1 mL buffer was added when the suspension in the sorting tube quickly drained out, and all the outgoing suspension was collected, that is, unlabeled pre-enriched CD56+ CD16-NK cells.

CD56+ CD16-NK cells obtained by magnetic sorting: The cells were centrifuged at 300×g for 10 min, the supernatant was discarded, 500 μL buffer was added for re-suspension, and 100 μL CD56 MicroBeads were added, mixed and incubated in the refrigerator at 4°C for 15 min away from light. Add 5 mL buffer buffer to wash the cells, centrifuge at low temperature and high speed for 300 g×10 min, and discard the supernatant. Add 500 μL buffer for suspension. Place the MS post in the magnetic field of the MACS separator and flush the post with 500 μL buffer buffer to prepare. The cell suspension was added to the column, unlabeled cells that passed through the column were collected, and the column was washed with a 3× 500μL buffer. The MS sorting column was removed from the magnetic field environment and placed in another EP tube. 1 mL buffer was added to the MS sorting column and the buffer was quickly squeezed into the EP tube with a piston. The resulting cell suspension was CD56+ CD16-NK cells with magnetic labels

CD56^{bright}NK culture

CD56^{bright}NK cells were cultured with IL-2 (final concentration: 100 U/mL) and IL-15 (final concentration: 100 U/mL). 10 ng/mL), penicillin (final concentration 100 U/mL), streptomycin (final concentration 100 μg/mL) and 5% exterminated human serum X-VIVO 10 medium, 80 μL old culture base was taken every three days, and 100 μL fresh medium containing cytokines was added into the hole. It can be used in experiments after 7 days of culture.

Plasmid construct and transfection

The plasmid extraction kit was purchased from Takara Company (Takara, Japan). The pHelper 1.0, pHelper 2.0 helper plasmids, and transfection reagents were purchased from GENE Company (Shanghai, China) For the full-length coding sequence of STAB1, design specific primers: Forward 5 ’- GGAATTCCTCTGTCCTGGACAGCGT - 3’; Reverse 5’- CTCTAGAACATGGCCCACATCCG -3’. The plasmid for STAB1 overexpression was transfected into 293T cells. The supernatant of 293T cells that had been transfected for 48 hours was collected and concentrated. The virus preservation liquid was added, fully dissolved, centrifuged, and then the supernatant was obtained. The viral titer was detected through RT-PCR. The culture medium of HBMEC cells was replaced with a Polybrene-containing medium. According to the MOI value, the appropriate volume of lentivirus was added to each well. Then, the transfection efficiency was verified using the Western Blot experiment.

Western Blot

Each group was prepared as a cell suspension and separately inoculated into 6-well plates at a cell number of 5×105 per well. When the cell fusion rate reached 90%, the experimental groups were treated according to the experimental design. Protein quantification was performed using the BCA protein quantification kit (KeyGEN Bio TECH, NanJing, China). SDS-PAGE gels were prepared according to the molecular weight of the target protein. After the gel was set, it was placed in an electrophoresis tank containing electrophoresis buffer. 5 μL of marker and sample were added to each well, with a loading volume of 60 μg. The gel was electrophoresed at 80 V for about 30 minutes, and the voltage was adjusted to 120 V after the sample entered the separation gel. The PVDF membrane was cut, activated in methanol for 1 minute, and then soaked in transfer buffer for 15 minutes. The filter paper was also soaked in the transfer buffer for 15 minutes. The membrane was stained with Fast Red S dye solution, followed by two TBST (G0001, Servicebio, WuHan, China) washes. The one-antibody was diluted with TBST to an appropriate concentration and incubated at 4°C overnight. An appropriate concentration of the secondary antibody was added and incubated at room temperature for 1 hour. The membrane protein was allowed to come into contact with the luminescent reagent for 5 minutes. The Tanon 5200 luminescence imaging workstation was used for detection. The protein expression level was analyzed by Image Pro Plus 6.0 software based on the optical density values. The relative expression level of the target protein was calculated as the gray value of the target protein/the gray value of the internal control protein. The following antibodies were used: Anti-STAB1

antibody (sc-293254, Santa Cruz Biotechnology, Inc, Texas, USA)Anti-β-actin antibody (ab6276, Abcam, Cambridge, UK).

Tube formation assay

The Matrigel gel was thawed overnight at 4°C in the refrigerator after being taken from the -20°C freezer the day before the experiment. The tips of the pipettes used in the experiment were placed in the -20°C freezer the day before and left there for pre-cooling. They were taken out 30 minutes before the experiment and placed on ice. In a 96-well plate, add 50 μL of melted Matrigel gel to each well without generating bubbles. Gently shake to level the gel and incubate at 37°C for 30 minutes to allow the Matrigel gel to fully solidify. Take the cell fusion rate at 70%-80%, digest the HBMEC cells in a trypsin solution for 3 minutes at 37°C, centrifuge for 3 minutes, resuspend in complete culture medium, and count the cells to determine the density of 1.5×104 cells/well. Do not touch the gel surface and gently blow the cell suspension to avoid large differences in cell numbers within each well. Use a microscope to observe whether the cells are distributed evenly. Gently shake the 96-well plate until the cells are evenly distributed. Set up three replicates per well and incubate at 37°C in a standard culture incubator for 6 hours; Closely observe the angiogenesis state after 2 hours of culture, and remove the 96-well plate when the lumen is fully formed after 6 hours of culture. Take photos under a 100×microscope. Analyze the total length of the microvilli using the ImageJ software, repeating the experiment three times.

Cell scratch assay

Use a UV-sterilized marker pen with evenly drawn horizontal lines behind the 6-well plate. Cells in the logarithmic phase were digested with trypsin to form a single cell suspension. Appropriate culture medium was added to adjust the concentration of the cell suspension, and the cells were seeded into 6-well plates with lines drawn on them. The total culture medium volume in each well was 2 mL in the end; Cultured for 24 hours at 37°C and 5% CO2 in a cell culture incubator. 4 hours later, observe the cells adhering under a microscope. In a clean bench, use a sterilized 200 μL pipet tip to make a vertical incision along the marked horizontal line behind. Wash the cells with PBS three times. Remove the cells that were scratched. Add serum-free culture medium and incubate at 37°C in a 5% CO2 incubator for culture. Take 100 microscopic photographs to ensure that the scratch is centered and perpendicular.

Cell proliferation ability detected by CCK8

The CCK-8 kit (C0038, Beyotime, ShangHai, China) is used to detect the cell proliferation ability. Centrifuge the cells to collect them, and then seed them at a density of 1×104/well into 96-well plates. Set up 3 replicate wells for each group, and add the corresponding culture medium according to the grouping. After incubating the cells for the specified time, the cell viability was determined by CCK-8 assay. Remove the old culture medium and wash the wells with PBS 3 times. Add 100 μL of culture medium containing 10% CCK-8 working solution to each well. Place the wells in a cell culture incubator for incubation. After incubating for 2 hours, the absorbance values of each group were detected at 450 nm by an enzyme-linked immunosorbent assay (ELISA) reader.

Immunofluorescence staining

Soak a cover slip (22x22 mm) in 75% alcohol for 2 hours, dry the alcohol, and place it in a 6-well plate. According to the experimental group, the cells were seeded into 6-well plates. They were cultured at 37°C and 5% CO2; When the cells had proliferated to 80%, the supernatant was aspirated. Add 500 μL of 4% formaldehyde for 15 min to fix the tissue. Wash with PBS 3 times, each time for 5 min. Add 0.1% TritonX-100, incubate at room temperature for 10 minutes. Wash with PBS 3 times, each time for 5 minutes; Block the cells with a blocking solution at room temperature for 30 minutes; Discard the blocking solution, add the primary antibody, and incubate overnight at 4°C. After incubation, remove and wash with PBS liquid 3 times, each time for 5 minutes; Add fluorescently labeled secondary antibody and incubate in the dark for 1 hour.
Wash with PBS 3 times, each time for 5 minutes; Add DAPI for mounting, suck out the excess liquid; Observed under a fluorescence microscope, 400x random selected fields were photographed. The following antibodies were used:： Actin-Tracker Green-488(C2201S, Beyotime, ShangHai, China); Vinculin Monoclonal antibody (66305-1-Ig, Proteintech, WuHan, China);CoraLite594–conjugated Goat Anti-Mouse IgG (H+L) (SA00013-3, Proteintech, WuHan, China)

Flow cytometry detects cell cycle

The cell cycle kit (KGA512, KeyGEN Biotech, NanJing, China) is used to detect the cell cycle. After the cells in each group were incubated according to the experimental

requirements, the lower layer was removed and the culture medium was discarded. Wash the cells with 0.5 mL of PBS twice. Discard the PBS. Add 2 mL of 0.25% trypsin solution without EDTA to digest the cells. Add 2 mL of complete culture medium to stop the digestion and collect the cells. Centrifuge at 800 rpm for 5 minutes at 4°C, and discard the supernatant. Suspend the cells in 500 μL of cold ethanol at 70% v/v. Collect fixed cells at 800 rpm for 15 minutes, and wash with PBS twice. Suspend the cells in 0.4 mL PBS and transfer them to a tube by gently pipetting (to prevent cell lysis). Add RNase-A approximately 3 μL to a final concentration of 50 μg/mL, and incubate at 37°C in a water bath for 30 minutes; Add PI at a final concentration of approximately 65 μg/mL for 30 minutes at 4°C in the dark. Filtered with a 300-mesh (pore size of 40-50 microns) nylon net and tested on the machine; Sample analysis and determination.

CFSE detects cell proliferation

Collect ex vivo-cultured Treg and $CD56^{bright}$NK cells. Centrifuge at 1500 rpm for 5 min, discard supernatant; Suspend the cells in room temperature PBS, centrifuge at 1500 rpm for 5 minutes, and discard the supernatant. Adjust the cell concentration to 1×107 cells/mL with room temperature PBS and add the CFSE (C34554, Thermo Fisher Scientific, Pittsburgh, PA, USA) dye (5μM). Incubate in a cell culture incubator for 8 minutes; Add 5 times the volume of culture medium to stop the staining, centrifuge at 1500 rpm for 5 minutes, discard the supernatant; Count the cells, and place them into 96-well plates. After incubation, collect the cells. The flow cytometer detects the fluorescence intensity of the CFSE-labeled cells.

Statistical analysis

Data were analyzed and plotted using GraphPad Prism 9 (Version 9.4.0), and organized and combined into figures using Adobe Illustrator 2022 (Version 26.3.0). All data were expressed as mean ± SD. Statistical differences between groups were analyzed using one-way ANOVA, and P values less than 0.05 were considered statistically significant.

Supplementary Figure

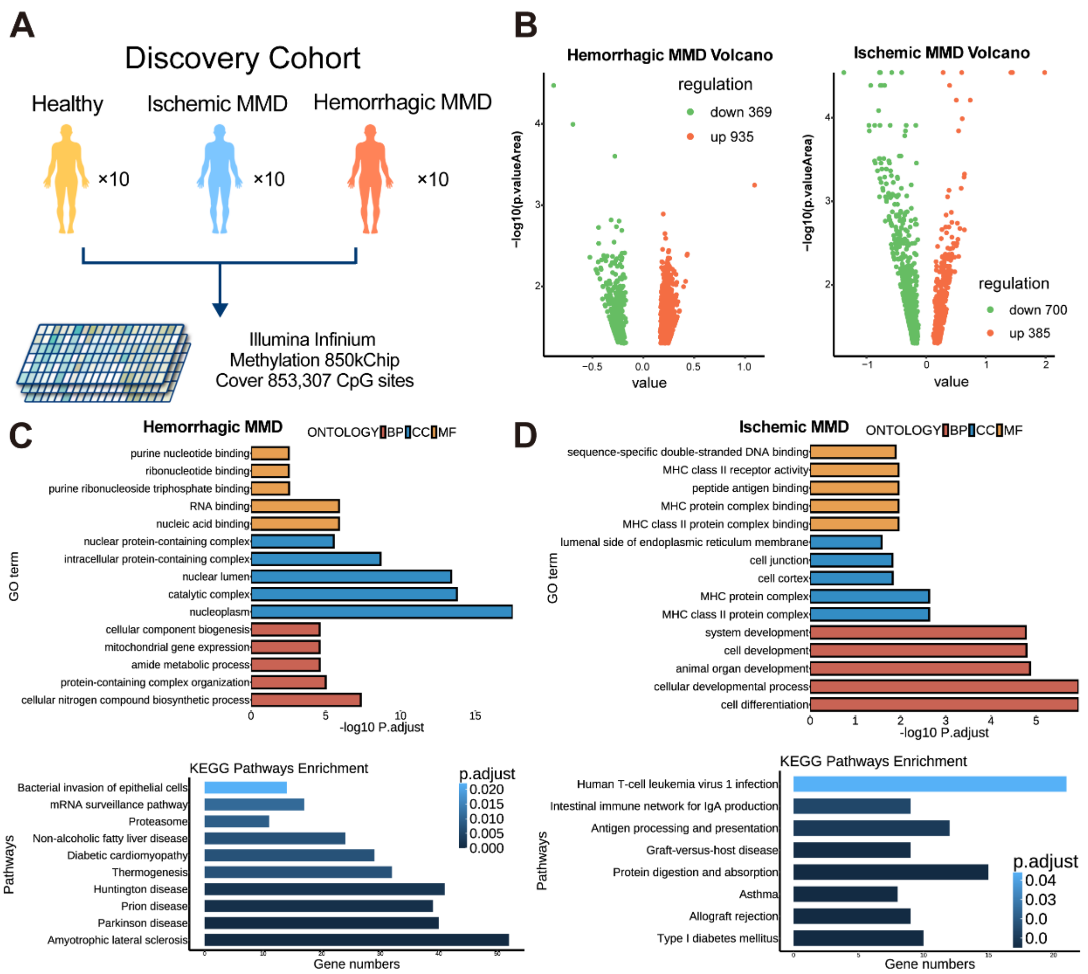

Figure S1: Analysis of DNA Methylation Profile in Moyamoya Disease

A: The discovery cohort underwent DNA methylation analysis by using the Illumina 850K chip.
B: The volcano plot presents the differentially methylated regions (DMRs) of Moyamoya Disease. Green indicates down-regulated DMRs, and orange indicates up-Regulated DMRs. The hemorrhagic Moyamoya Disease has 935 hypermethylated DMRs and 369 hypomethylated DMRs, while the ischemic Moyamoya Disease has 385 hypermethylated DMRs and 700 hypomethylated DMRs.
C: The bar chart displays the GO and KEGG enrichment analysis of DMG in hemorrhagic Moyamoya Disease. The vertical axis represents the enriched items. Orange represents BP items. Blue represents CC items. Red represents MF items.
D: The bar chart exhibits the GO and KEGG enrichment analysis results of DMG in ischemic Moyamoya Disease. The vertical axis represents the enriched items. Orange represents BP items. Blue represents CC items. Red represents MF items.

（Moyamoya disease：MMD；Hemorrhagic MMD [HEM]=10；Ischemic MMD=10；

Healthy controls [HC]=10； BP： biological process； CC： cellular component; MF: molecular function)

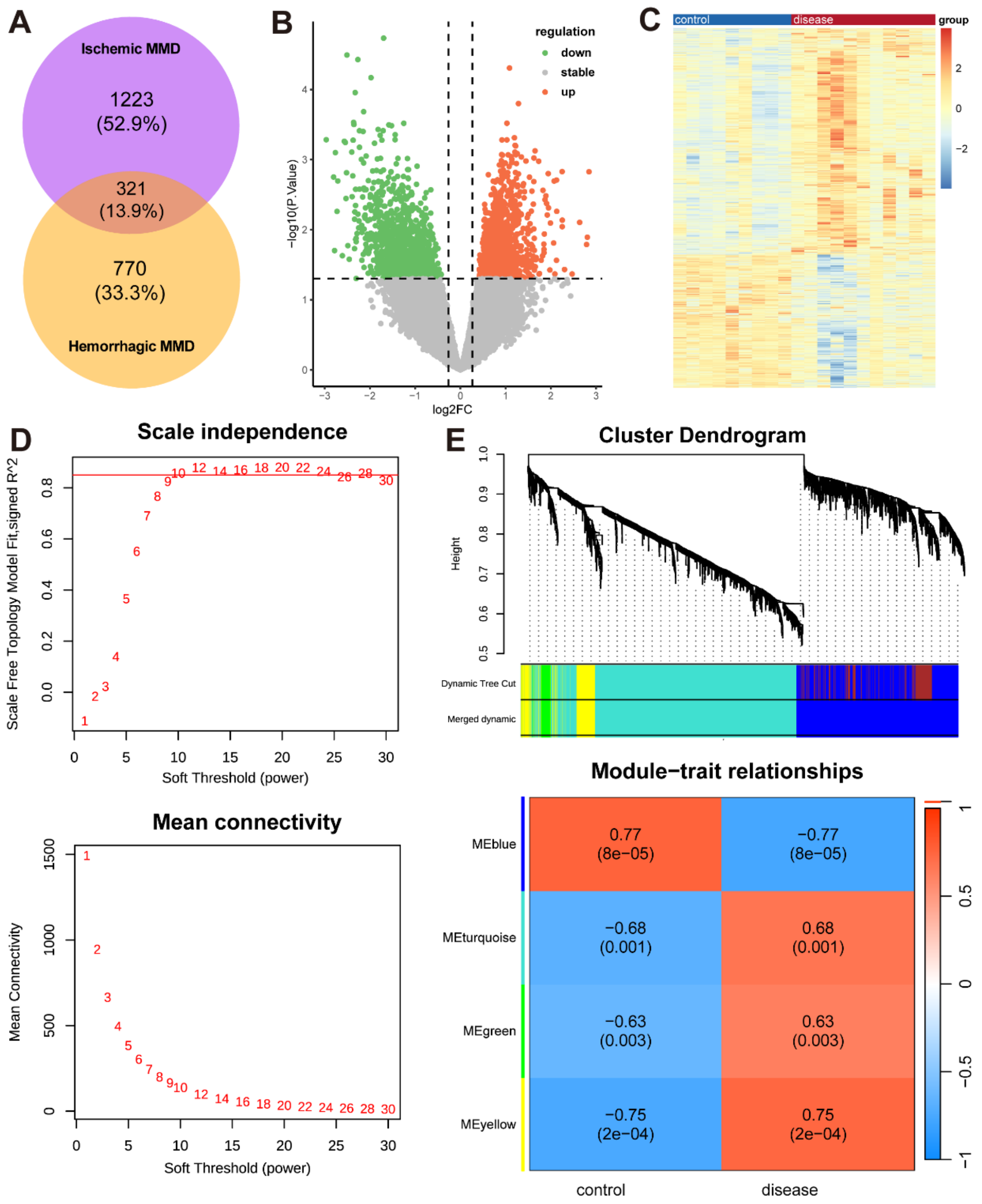

Figure S2: Identification of methylation-related differential disease hub genes in MMD

A: The Venn diagram depicts the intersection of differentially methylated genes (DMGs) in case A (hemorrhagic moyamoya disease) and case B (ischemic moyamoya disease), with a total of 321 moyamoya disease differentially methylated genes being obtained.

B: The volcano plot reveals that a total of 1,797 up-regulated differential genes and 1,053 down-regulated differential genes were acquired after differential analysis was carried out on the validation cohort.

C: The heat map exhibits the expression profiles of the top 50 up-regulated and down-regulated genes subsequent to differential analysis being conducted on the validation cohort. The x-axis: blue represents the control group, and red represents the disease group. Red indicates up-regulated genes, and blue indicates down-regulated genes.

D: When the soft power threshold was set to 10, the Scale free topology model fit signed R^2 was above 0.85, and the Mean connectivity approximated 0.

F: Setting the minimum number of genes for each gene module at 50 resulted in 5 modules. Three of the modules, namely turquoise, green, and yellow, have a relatively higher correlation with moyamoya disease ($r > 0.6$, $p < 0.05$). The highest correlation is with the yellow module.

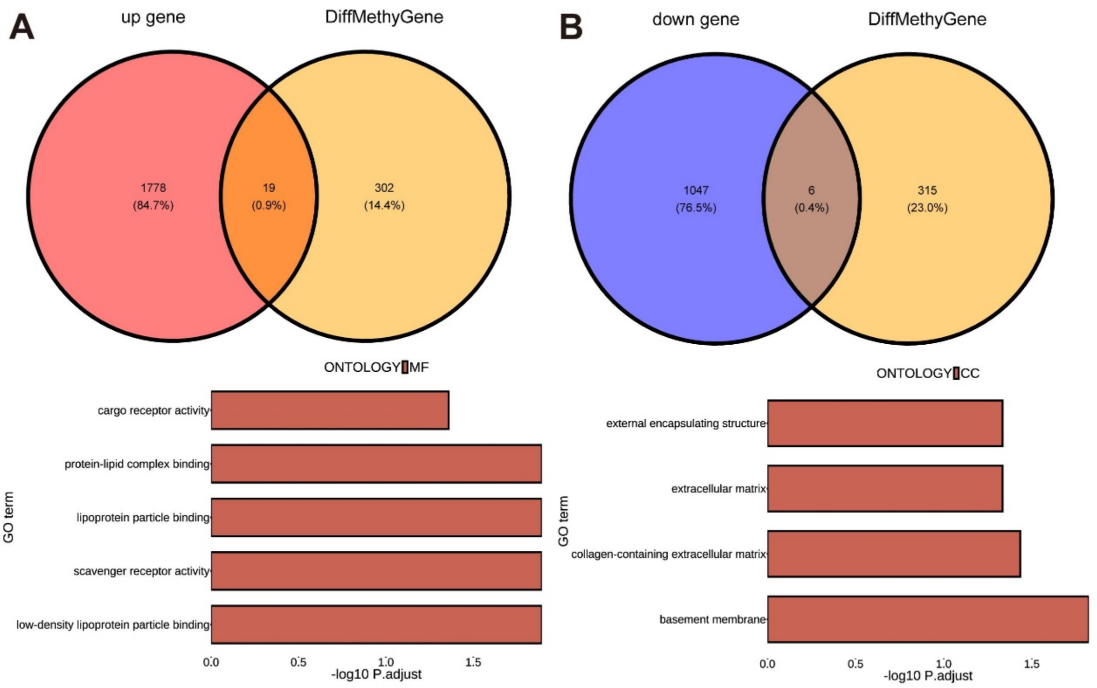

Figure S3: Selection and GO enrichment analysis of differentially methylated genes related to moyamoya disease.

(A) Venn diagram shows the intersection of upregulated differential genes and differentially methylated genes in moyamoya disease.

Bar chart shows the GO enrichment analysis results of the intersection genes. MF: Molecular function. (B) Venn diagram shows the intersection of downregulated differential genes and differentially methylated genes in moyamoya disease.

Bar chart shows the GO enrichment analysis of the intersection genes. CC: Cellular component.

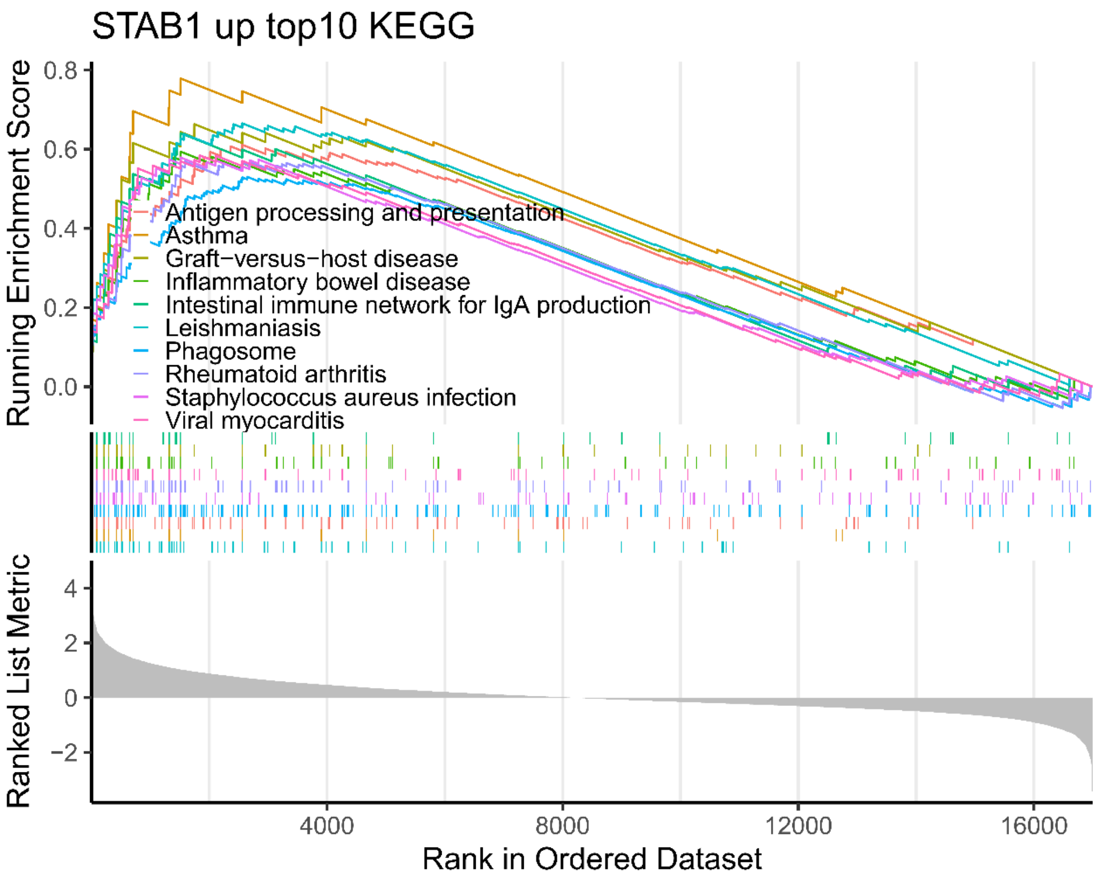

Figure S4: GSEA analysis of STAB1 reveals the top ten upregulated pathways.

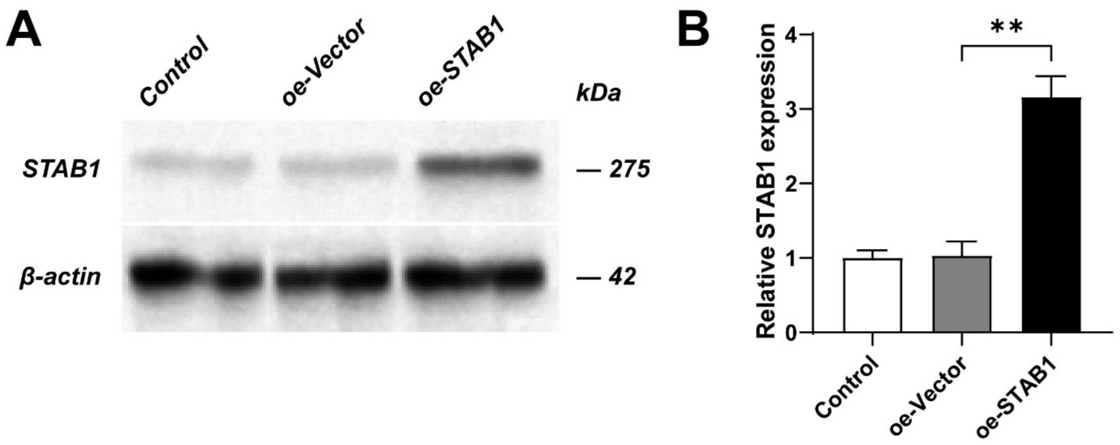

Figure S5 Detection of plasmid transfection efficiency.

(A) Western Blot detects the efficiency of plasmid transfection. (B) A bar chart shows the expression level of STAB1 in HBMEC after transfection with the plasmid.

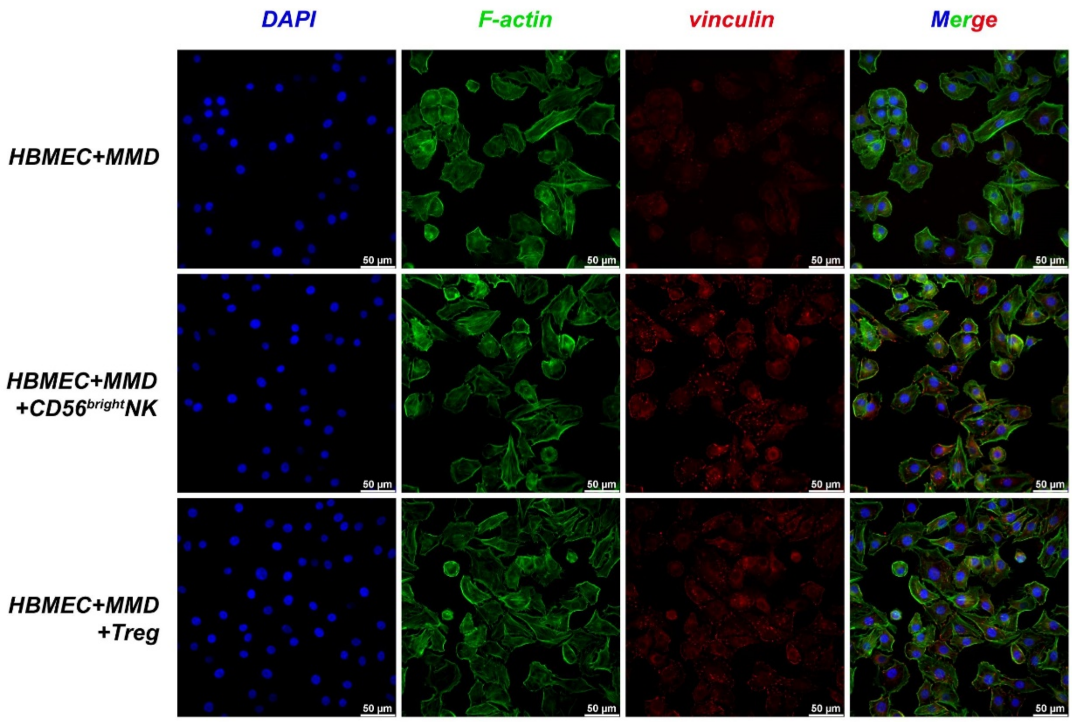

Figure S6 Immunofluorescence staining of HBMEC after serum-stimulated culture and co-culture. Blue (DAPI): nucleus. Green (F-actin): cytoskeleton. Red (vinculin): adherens junction. Scale bar: 50 μm

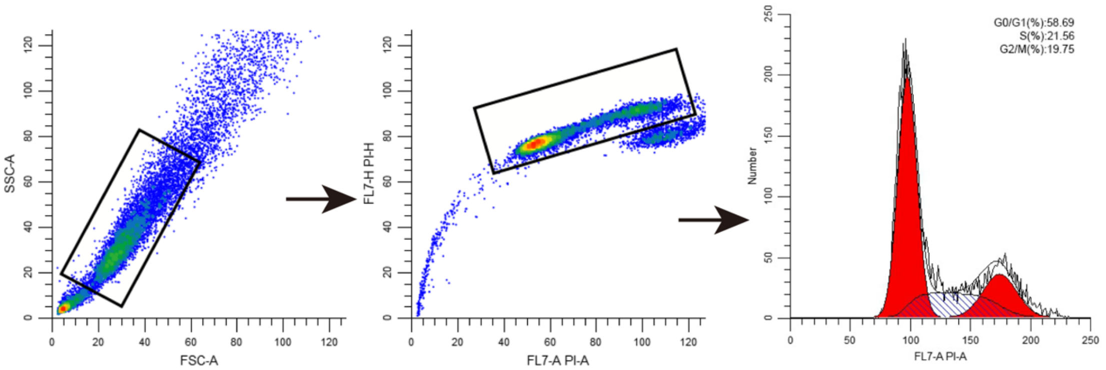

Figure S7 Gating strategy schematic for the cell cycle analysis

Cells were gated on FSC-A/SSC-A to exclude cell debris. Gate on the single cell population using FL7-H PI-H h vs. FL7-A PI-A. Then apply this gate to the scatter plot and gate out obvious debris. Combine the gates and apply to the PI histogram plot.

Table S1: Clinical characteristics and sample information of the patients in discovery cohort.

No. of Patients	Groups	Sex	Age (years)	Hypertension	Diabetes	Coronary heart disease	Hyperlipidemia	Smoking history	Alcohol taking	Duration of symptoms (months)	Suzuki stage	Family history
1	HEM	M	32	NO	NO	NO	NO	NO	NO	5	L5/R4	NO
2	HEM	F	42	NO	NO	NO	NO	NO	NO	13	L2/R3	NO
3	HEM	M	23	NO	NO	NO	NO	NO	NO	4	L3/R2	NO
4	HEM	F	50	NO	NO	NO	NO	NO	NO	5	L3/R1	NO
5	HEM	F	26	NO	NO	NO	NO	NO	NO	14	L5/R1	NO
6	HEM	M	32	NO	NO	NO	NO	NO	NO	34	L4/R5	NO
7	HEM	F	43	NO	NO	NO	NO	NO	NO	11	L3/R2	NO
8	HEM	M	27	NO	NO	NO	NO	NO	NO	5	L1/R1	NO
9	HEM	F	34	NO	NO	NO	NO	NO	NO	6	L3/R3	NO
10	HEM	M	18	NO	NO	NO	NO	NO	NO	2	L4/R3	NO
11	IS	F	46	NO	NO	NO	NO	NO	NO	4	L1/R2	NO
12	IS	F	47	NO	NO	NO	NO	NO	NO	11	L3/R6	NO
13	IS	M	37	NO	NO	NO	NO	NO	NO	5	L3/R3	NO
14	IS	M	46	NO	NO	NO	NO	NO	NO	5	L2/R4	NO
15	IS	F	16	NO	NO	NO	NO	NO	NO	7	L2/R3	NO
16	IS	M	25	NO	NO	NO	NO	NO	NO	4	L2/R1	NO
17	IS	M	43	NO	NO	NO	NO	NO	NO	22	L2/R2	NO
18	IS	F	52	NO	NO	NO	NO	NO	NO	34	L3/R2	NO

19	IS	F	28	NO	NO	NO	NO	NO	NO	44	L3/R2	NO
20	IS	M	41	NO	NO	NO	NO	NO	NO	12	L5/R3	NO
21	HC	F	23	NO	NO	NO	NO	NO	NO	NO	NO	NO
22	HC	F	29	NO	NO	NO	NO	NO	NO	NO	NO	NO
23	HC	F	49	NO	NO	NO	NO	NO	NO	NO	NO	NO
24	HC	M	25	NO	NO	NO	NO	NO	NO	NO	NO	NO
25	HC	M	29	NO	NO	NO	NO	NO	NO	NO	NO	NO
26	HC	F	39	NO	NO	NO	NO	NO	NO	NO	NO	NO
27	HC	F	33	NO	NO	NO	NO	NO	NO	NO	NO	NO
28	HC	M	43	NO	NO	NO	NO	NO	NO	NO	NO	NO
29	HC	M	34	NO	NO	NO	NO	NO	NO	NO	NO	NO
30	HC	M	39	NO	NO	NO	NO	NO	NO	NO	NO	NO

MMD, moyamoya disease; HEM, hemorrhagic moyamoya disease; IS, ischemic moyamoya disease. M, Male; F, Female. L, left; R, right.; Duration of symptoms indicates the duration from the first symptom until the hospitalization. The discovery cohorts of this study were all Han Chinese.

Table S2: Clinical characteristics and sample information of the patients in validation cohort (GSE157628).

ID	disease	Age(year)	sex	Taken medicines	Antibiotics for the operation	Clinical presentation	Duration until surgery from the last clinical presentation	Subtype of MMD	Aneurysm location/size	Epileptic origin
1	MMD	64	F	Atorvastatin 10 mg/day	Cefazolin 1 g	ICH	2 months	Bilateral	NO	NO
2	MMD	47	F	Aspirin 100 mg/day, atorvastatin 10 mg/day	Ceftriaxone 2 g	TIA	5 months	Bilateral	NO	NO
3	MMD	48	F	Amlodipine 5 mg/day	Ceftriaxone 2 g	ICH	6 months	Bilateral	NO	NO
4	MMD	49	F	Aspirin 100 mg/day	Cefazolin 1 g	TIA	6 months	Bilateral	NO	NO
5	MMD	59	F	Cilostazol 200 mg/day	Cefazolin 1 g	IF	11 months	Bilateral	NO	NO
6	MMD	50	F	Cilostazol 200 mg/day	Cefazolin 1 g	IF	8 months	Bilateral	NO	NO
7	MMD	45	F	Aspirin 100 mg/day, clopidogrel 75 mg/day	Clindamycin 600 mg	TIA	1 month	Bilateral	NO	NO
8	MMD	48	F	Aspirin 100 mg/day	Cefazolin 1 g	TIA	4 months	Bilateral	NO	NO
9	MMD	51	M	Aspirin 100 mg/day, olmesartan 20 mg/day, azelnidipine 16 mg/day, pitavastatin 2 mg/day	Cefazolin 1 g	TIA	2 months	Bilateral	NO	NO

10	MMD	53	F	Aspirin 100 mg/day	Cefazolin 1 g	TIA	4 months	Bilateral	NO	NO
11	MMD	43	F	Aspirin 100 mg/day, telmisartan 80 mg/day	Cefazolin 1 g	TIA	10 months	Bilateral	NO	NO
12	IA	62	F	NO	Cefazolin 1 g	NO	NO	NO	Supraclinoid/ 27 mm	NO
13	IA	78	F	NO	Cefazolin 1 g	Oculomotor nerve palsy	5 months	NO	Cavernous/ 30 mm	NO
14	IA	79	F	Cilostazol 100 mg/day, candesartan 8 mg/day, benidipine 4 mg/day, rosuvastatine 5 mg/day, ezetimibe 10 mg/day	Cefmetazole 1 g	Oculomotor nerve palsy	6 months	NO	Cavernous/ 25 mm	NO
15	IA	70	F	Clopidogrel 75 mg/day, valsartan 160 mg/day, amlodipine 10 mg/day, imidapril 5 mg/day, doxazosin 2 mg/day, methotrexate 10 mg/week	Cefazolin 1 g	Oculomotor nerve palsy	7 months	NO	Cavernous/ 20 mm	NO
16	IA	65	F	Clopidogrel 75 mg/day, atorvastatin 10 mg/day	Cefazolin 1 g	NO	NO	NO	Cavernous/ 26 mm	NO
17	IA	71	F	Aspirin 100 mg/day, valsartan 160 mg/day, amlodipine 5 mg/day	Cefazolin 1 g	NO	NO	NO	Supraclinoid/ 22 mm	NO
18	EPI	56	M	Pravastatin 20 mg, levetiracetam 2000 mg/day, lamotrigine 300	Cefazolin 1 g	Seizures	Several days	NO	NO	Temporal lobe

				mg/day, perampanel 4 mg/day						
19	EPI	14	M	Lacosamide 300 mg/day, perampanel 6 mg/day, clobazam 15 mg/day	Cefazolin 1 g	Seizures	Several days	NO	NO	Frontal lobe
20	EPI	20	M	Lacosamide 300 mg/day, lamotrigine 200 mg/day, carbamazepine 400 mg/day	Cefazolin 1 g	Seizures	Several days	NO	NO	Frontal lobe

MMD, moyamoya disease; F, female; M, male; IA, intracranial aneurysm; EPI, epilepsy; ONP, ocular nerve palsy; ICH, intracerebral hemorrhage; IF, infarction; TIA, transient ischemic attack; ICA, internal carotid artery; MCA, middle cerebral artery;
